# Identification of *Mycobacterium abscessus* using the peaks of ribosomal protein L29, L30 and hemophore-related protein by MALDI-MS proteotyping

**DOI:** 10.1038/s41598-024-61549-7

**Published:** 2024-05-16

**Authors:** Satomi Takei, Kanae Teramoto, Yuji Sekiguchi, Hiroaki Ihara, Mari Tohya, Shinichi Iwamoto, Koichi Tanaka, Abdullah Khasawneh, Yuki Horiuchi, Shigeki Misawa, Toshio Naito, Teruo Kirikae, Tatsuya Tada, Yoko Tabe

**Affiliations:** 1 Department of Clinical Laboratory Medicine, Juntendo University Graduate School of Medicine, Tokyo, Japan; 2 Department of MALDI-TOF MS Practical Application Research, Juntendo University Graduate School of Medicine, Tokyo, Japan; 3 Analytical and Measurement Instruments Division, Shimadzu Corporation, Kyoto, Japan; 4 Biomedical Research Institute, National Institute of Advanced Industrial Science and Technology (AIST), Tsukuba, Ibaraki Japan; 5 Department of Respiratory Medicine, Juntendo University Graduate School of Medicine, Tokyo, Japan; 6 Department of Microbiology, Juntendo University Graduate School of Medicine, 2-1-1 Hongo, Bunkyo-ku, Tokyo, 113-8421 Japan; 7 Koichi Tanaka Mass Spectrometry Research Laboratory, Shimadzu Corporation, Kyoto, Japan; 8 Department of Clinical Laboratory Technology, Faculty of Medical Science, Juntendo University, Tokyo, Japan; 9 Department of General Medicine, Juntendo University Graduate School of Medicine, Tokyo, Japan; 10 Department of Microbiome Research, Juntendo University Graduate School of Medicine, Tokyo, Japan

**Keywords:** Microbiology, Clinical microbiology

## Abstract

*Mycobacteroides* (*Mycobacterium*) *abscessus*, which causes a variety of infectious diseases in humans, is becoming detected more frequently in clinical specimens as cases are spreading worldwide. Taxonomically, *M. abscessus* is composed of three subspecies of *M. abscessus* subsp. *abscessus*, *M. abscessus* subsp. *bolletii*, and *M. abscessus* subsp. *massiliense,* with different susceptibilities to macrolides. In order to identify rapidly these three subspecies, we determined useful biomarker proteins, including ribosomal protein L29, L30, and hemophore-related protein, for distinguishing the subspecies of *M. abscessus* using the matrix-assisted laser desorption/ionization mass spectrometry (MALDI-MS) profiles. Thirty-three clinical strains of *M. abscessus* were correctly identified at the subspecies-level by the three biomarker protein peaks. This study ultimately demonstrates the potential of routine MALDI-MS-based laboratory methods for early identification and treatment for *M. abscessus* infections.

## Introduction

Members of the *Mycobacteroides* (*Mycobacterium*) *abscessus* cause various infectious diseases in humans that are spreading worldwide, including infections of the lungs, lymph nodes, skin, soft tissue, and bone^1–3^. However, treatment for *M. abscessus* infections are difficult due to their natural multidrug resistance^4^. The American Thoracic Society (ATS) and Infectious Disease Society of America (IDSA) recommended multidrug therapy based on macrolide in their 2007 guidelines^5–7^. Notably, the 2020 guideline from ATS, European Respiratory Society (ERS), European Society of Clinical Microbiology and Infectious Disease (ESCMID), and IDSA recommended the choice of antibiotics depending on the presence of the 23S rRNA methylase encoding gene, *erm* (41), and mutations in the 23S rRNA (*rrl*) gene^8,9^.

*M. abscessus* has been taxonomically reclassified many times and is now composed of three subspecies groups: *M. abscessus* subsp. *abscessus*, *M. abscessus* subsp. *bolletii*, and *M. abscessus* subsp. *massiliense*^10,11^. Members of the *M. abscessus* subsp. *abscessus* and *M. abscessus* subsp. *bolletii* are known to be mostly resistant to macrolide because these subspecies produce the 23S rRNA methylase Erm (41). On the other hand, *M. abscessus* subsp. *massiliense* is known to be relatively susceptible to macrolide because susceptible strains have a frameshift mutation in the 23S rRNA methylase Erm (41)^12^. Therefore, it is important to distinguish *M. abscessus* strains at the subspecies-level in treatment^13,14^.

In general, *M. abscessus* can be characterized at the subspecies-level by sequencing genes relevant to phylogeny and antibiotic resistance, such as 16S rRNA, *erm* (41), *hsp65*, and *rpoB*^15,16^. However, sequencing such genes is complicated and time-consuming. To solve these problems, GenoType NTM DR (Bruker Daltonik, Germany) was developed in 2016^17^. The GenoType NTM DR is a PCR-based test capable of identifying *M. abscessus* subspecies and detecting clarithromycin or amikacin resistance without sequencing 5 h^18^.

Matrix-assisted laser desorption/ionization mass spectrometry (MALDI-MS) can rapidly measure the mass of intact microbial constituents, such as proteins, with minimal sample pretreatment and can be used for the identification of microorganisms in the clinical laboratory on a routine basis^19^. The MALDI-MS-based method is considered rapid and highly accurate for the identification of species including rapidly growing mycobacterium (RGM) in clinical laboratories^20^. On the other hand, commercially available MALDI-MS microbial identification systems, such as Biotyper (Bruker Daltonik) and VITEK-MS (bioMerieux, France), have been exclusively designed for the identification of strains at the genus or species level, but it is difficult to distinguish the subspecies using these systems. Previous studies report that the three subspecies of *M. abscessus* can be distinguished by subspecies-specific MS peaks using the principal component analysis and machine learning of MALDI-MS^20,21^, but the biomarker peaks for some isolates of *M. abscessus* subsp. *abscessus* and *M. abscessus* subsp. *massiliense* are too similar and therefore hard to distinguish at the subspecies-level^22–26^.

In this study, we developed new criteria for the MALDI-MS proteotyping of *M. abscessus,* allowing for subspecies-level discrimination. Potential biomarker mass peaks were carefully selected in MALDI-MS measurements using cultures including all *M. abscessus* subspecies. Corresponding protein sequences of these peaks were inferred from the masses of the proteins theoretically encoded in the genome of each type strain. We newly detected some strong mass peaks that characterize the strains of *M. abscessus* at the subspecies-level. The detection of these peaks was then used as criteria for characterizing 33 clinical *M. abscessus* isolates at the subspecies-level. The present study contributes to the exact subspecies identification of *M. abscessus* strains in routine microbiological examinations and paves the way for early determination of treatment strategies.

## Results

### Clinical features and drug-resistant genes of *M. abscessus*

The 33 clinical strains of *M. abscessus* were isolated from 2013 to 2019 at Juntendo University Hospital. The whole-genomes of the 33 M*. abscessus* were sequenced using MiSeq. According to the latest method for distinguishing between different *M. abscessus* subspecies using average nucleotide identity (ANI), 16S rRNA, *rpoB*, *hsp65*, and *erm* genes^15,27^, we obtained clinical isolates of 22 *M. abscessus* subsp. *massiliense* and 11 *M. abscessus* subsp. *abscessus.* There were no clinical isolates of *M. abscessus* subsp. *bolletii* in this study. The detailed information of ANI, 16S rRNA, *rpoB*, *hsp65*, and *erm* genes are shown in Table S1. The results indicate that the *M. abscessus* subspecies cannot be distinguished using ANI or sequence of each gene.

The susceptibilities of these isolates to various antibiotics were tested using the microdilution method as described by the guidelines of the Clinical and Laboratory Standards Institute^28^. MICs of clarithromycin were determined at an early reading time (ERT) and a late reading time (LRT) for detecting the inducible macrolide resistance. Among the 11 *M. abscessus* subsp. *abscessus* isolates, one isolate was resistant to clarithromycin at the ERT, 8 were resistant to clarithromycin at the LRT, 3 were resistant to imipenem, and all 11 were susceptible to amikacin. The 9 clarithromycin-resistant isolates at the ERT and/or LRT had T28 *erm* (41) sequevar, whereas the remaining 2 clarithromycin-susceptible isolates had C28 *erm* (41) sequevar (Supplementary S2).

Among the 22 *M. abscessus* subsp. *massiliense* isolates, 2 isolates were resistant to clarithromycin at both the ERT and LRT, 8 were resistant to imipenem, and all 22 were susceptible to amikacin (Table 1). The 2 clarithromycin-resistant isolates at the ERT and/or LRT had a point mutation in *rrl* gene with an A2058C substitution in its 23S rRNA (Supplementary Table S2).

**Table 1:** Drug susceptibility profiles in clinical isolates of *Mycobacterium abscessus* (N = 33).

Subspecies	Antibiotics reagent	Breakpoint for resistance (μg/ml)	No. of resistant isolates (%)	MIC (μg/ml)		
				Range	MIC_50_	MIC_90_
*M. abscessus* subsp. *abscessus* (N = 11)	Clarithromycin ERT*	≥ 8	9.1	0.0625–128	0.25	1
	Clarithromycin LRT**	≥ 8	72.7	0.0625–128	128	> 128
	Amikacin	≥ 64	0	4–16	8	16
	Imipenem	≥ 16	27.3	4–16	8	16
*M. abscessus* subsp. *massiliense* (N = 22)	Clarithromycin ERT*	≥ 8	9.1	0.0625–128	0.0625	0.0625
	Clarithromycin LRT**	≥ 8	9.1	0.0625–128	0.0625	0.25
	Amikacin	≥ 64	0	0.125–16	8	16
	Imipenem	≥ 16	36.4	0.0625–16	8	16

Breakpoints for antimicrobial resistance were determined according to CLSI guidelines.

*ERT: early reading time (reading on the 5th day).

**LRT: late reading time (reading on the 14th day).

Our study revealed that 72.7% of *M. abscessus* subsp. *abscessus* isolates were resistant to clarithromycin whereas 9.1% of *M. abscessus* subsp. *massiliense* isolates were resistant to clarithromycin.

### MALDI-MS from type strains of *M. abscessus*

**Figure 1:**
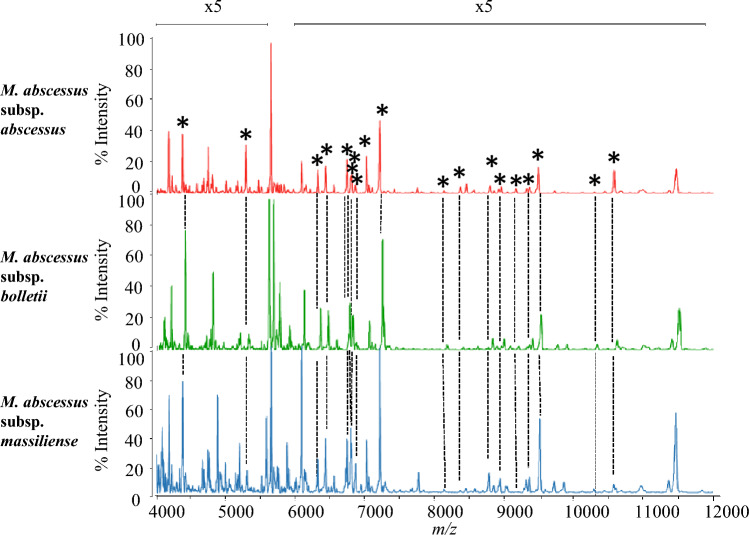
Representative mass spectra of *M. abscessus*. Red, green, and blue spectra are *M. abscessus* subsp. *abscessus* (ATCC 19977^T^), *M. abscessus* subsp. *bolletii* (JCM 15297^T^) and *M. abscessus* subsp. *massiliense* (JCM 15300^T^), respectively. Mass spectra and observed proteins from *m/z* 4000 to 12,000 were merged. The peaks marked with asterisks indicate the assigned peaks based on calculated masses within the tolerance at 500 ppm.

**Figure 2:**
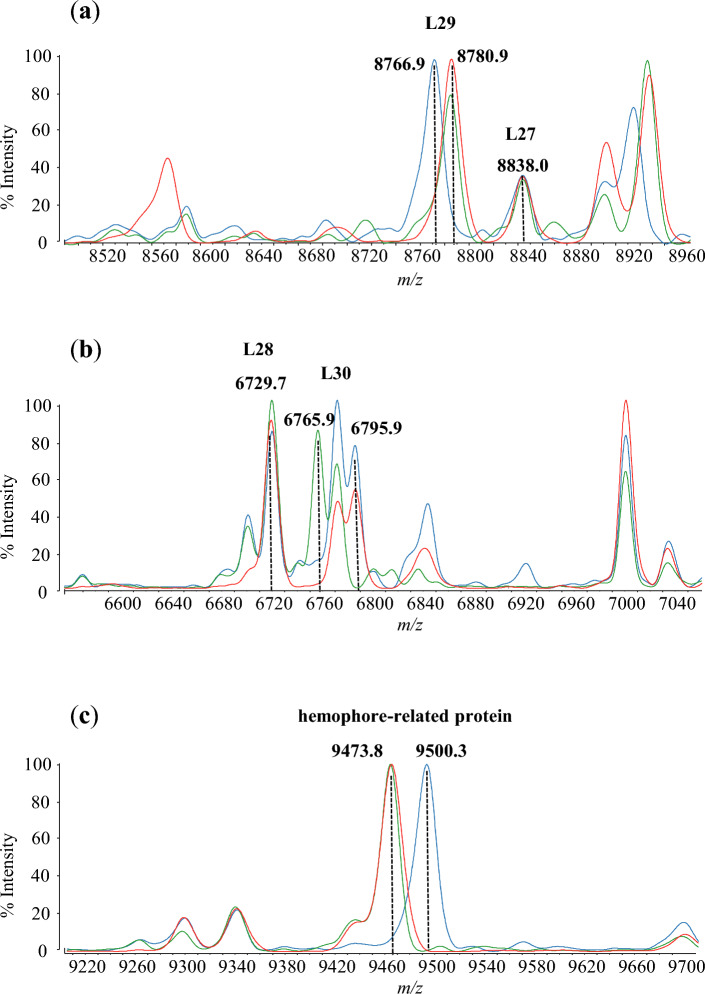
The peaks of ribosomal protein L27, L28, L29, L30, and hemophore-related protein are indicated in red for *M. abscessus* subsp. *abscessus* (ATCC 19977^T^), green for *M. abscessus* subsp. *bolletii* (JCM 15297^T^), and blue for *M. abscessus* subsp. *massiliense* (JCM 15300^T^). (**a**) The peaks of *m/z* 8766.9, *m/z* 8780.9, and *m/z* 8780.9 are represented as ribosomal protein L29 of *M. abscessus* subsp. *massiliense* (JCM 15300^T^) (blue), *M. abscessus* subsp. *abscessus* (ATCC 19977^T^) (red), and *M. abscessus* subsp. *bolletii* (JCM 15297^T^) (green), respectively. (**b**) The peaks *m/z* 6765.9, and *m/z* 6795.9 are represented as ribosomal protein L30 of *M. abscessus* subsp. *bolletii* (JCM 15297^T^) (green), *M. abscessus* subsp. *abscessus* (ATCC 19977^T^) (red), and *M. abscessus* subsp. *massiliense* (JCM 15300^T^) (blue), respectively. (**c**) The peaks *m/z* 9500.3, *m/z* 9473.8, and *m/z* 9473.8 are represented as hemophore-related protein of *M. abscessus* subsp. *massiliense* (JCM 15300^T^) (blue), *M. abscessus* subsp. *abscessus* (ATCC 19977^T^) (red), and *M. abscessus* subsp. *bolletii* (JCM 15297^T^) (green), respectively.

The observed peaks of the three *M. abscessus* subspecies using MALDI-MS are shown in Fig. 1. Table 2 summarizes the assigned proteins by MALDI-MS, together with the calculated and observed masses. Nineteen detected peaks were assigned with annotated proteins and reproducibility (Fig. 1; Table 2). Of these 19 annotated proteins, 14 proteins were ribosomal subunit proteins. The three biomarkers, including L29, L30 and hemophore-related protein, are able to distinguish the three subspecies of *M. abscessus* from the 19 annotated proteins. The peaks of *M. abscessus* subsp. *abscessus* and *M. abscessus* subsp. *bolletii* isolates were detected at *m/z* 8780.9 as ribosomal protein L29, whereas *M. abscessus* subsp. *massiliense* isolates were detected at *m/z* 8766.9. Moreover, the peaks of *M. abscessus* subsp. *abscessus* and *M. abscessus* subsp. *massiliense* isolates were detected at *m/z* 6795.9 as ribosomal protein L30, whereas *M. abscessus* subsp. *bolletii* isolates were detected at *m/z* 6765.9. Finally, the peaks of *M. abscessus* subsp. *abscessus* and *M. abscessus* subsp. *bolletii* isolates were detected at *m/z* 9473.8 as hemophore-related protein, whereas *M. abscessus* subsp. *massiliense* isolates were detected at *m/z* 9500.3 (Fig. 2). Analysis by MALDI-8020 and Microflex LT/SH revealed that the peaks of ribosomal protein L29, L30, and hemophore-related protein from the three subspecies cultured in 5% sheep blood agar (Becton, Dickinson-Diagnostic Systems, Sparks, MD, USA) were identical to those cultured in Middlebrook 7H11 Agar plates (Becton, Dickinson-Diagnostic Systems) (Supplementary Tables S3 and S4).

**Table 2:** Annotated peaks of type strains of *Mycobacterium abscessus.*

Biomarker proteins	Subspecies											
	*Mycobacterium abscessus* subsp. *abscessus* ATCC 19977^T^				*Mycobacterium abscessus* subsp. *bolletii* JCM 15297^T^				*Mycobacterium abscessus* subsp. *massiliense* JCM 15300^T^			
	Calculated masses (*m/z*)	Average	SE	Peak numbers (n = 5)	Calculated masses (*m/z*)	Average	SE	Peak numbers (n = 5)	Calculated masses (*m/z*)	Average	SE	Peak numbers (n = 5)
L36	4371.3	4372.0	0.18	5	4371.3	4369.8	0.37	5	4371.3	4373.0	0.36	5
L34	5460.4	5461.0	0.36	3	5460.4	5460.6	0.44	5	5460.4	5462.2	0.37	4
L33 2	6310.2	6310.8	0.24	5	6310.2	6310.7	0.43	5	6310.2	6311.1	0.40	5
L32	6423.5	6423.7	0.23	5	6423.5	6423.7	0.44	5	6423.5	6424.7	0.37	5
L28	6729.7	6729.6	0.16	5	6729.7	6730.6	0.39	5	6729.7	6731.2	0.42	5
L30	6795.9	6796.5	0.18	5	6765.9	6767.4	0.43	5	6795.9	6797.3	0.38	5
S14 type Z	6781.1	6782.3	0.17	5	6781.1	6782.3	0.40	5	6781.1	6782.9	0.25	5
Pup	6852.2	6853.6	0.24	5	6824.1	6825.8	0.38	5	6852.2	6854.9	0.33	5
L35	7092.2	7092.1	0.39	2	7092.2	7093.2	0.44	5	7092.2	7092.9	0.57	5
Probable cold shock protein A	7199.9	7201.0	0.17	5	7199.9	7201.8	0.42	5	7199.9	7202.1	0.33	5
L31	8123.2	8122.3	0.29	5	8123.2	8125.3	0.37	5	8123.2	8122.1	0.64	3
Translation initiation factor IF-1	8358.7	8358.4	0.28	5	8358.7	8361.9	0.48	5	8358.7	8359.0	0.86	2
L29	8780.9	8781.0	0.21	5	8780.9	8784.5	0.43	5	8766.9	8768.1	0.24	5
L27	8838.0	8838.7	NA*	1	8838.0	8842.6	0.77	5	8838.0	8840.1	0.48	5
S18 2	9153.7	9152.6	0.19	5	9153.7	9152.8	0.43	5	9153.7	NA	NA	0
S20	9348.8	9348.9	0.08	5	9348.8	9353.0	0.52	5	9348.8	9350.7	0.28	1
Hemophore-related protein	9473.8	9473.2	0.22	5	9473.8	9477.3	0.44	5	9500.3	9502.4	0.35	5
S15	10,279.0	NA	NA	0	10,279.0	10,283.9	0.58	5	10,279.0	10,281.2	0.47	5
10 kDa chaperonin	10,559.9	10,561.3	0.29	5	10,559.9	NA	NA	5	10,559.9	10,564.6	0.31	5

*Not assigned.

**Figure 3:**
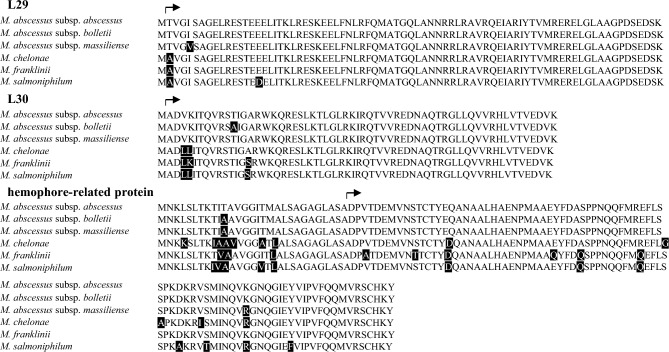
Amino acid sequence alignment of ribosomal protein L29, L30, and hemophore-related protein of *M. abscessus*, *M. chelonae*, *M. franklinii*, and *M. salmoniphilum*. Amino acid substitutions are shaded in black. The arrows indicate the start of amino acid residues after post-transcriptional modifications.

When compared with the amino acid sequences of these three proteins in *M. abscessus* subsp. *abscessus*, an amino acid substitution was observed at position 5 of L29 from Ile to Val (Ile5Val) in *M. abscessus* subsp. *massiliense,* at position 12 of L30 from Thr to Ala (Thr12Ala) in *M. abscessus* subsp. *bolletii,* and at position 90 of hemophore-related protein from Lys to Arg (Lys90Arg) in *M. abscessus* subsp. *massiliense* (Fig. 3). The amino acid substitutions of Ile5Val, Thr12Ala, and Lys90Arg were unique in *M. abscessus* subsp. *massiliense, M. abscessus* subsp. *bolletii,* and *M. abscessus* subsp. *massiliense,* respectively.

These results indicate that L29, L30, and hemophore-related protein can be biomarkers to distinguish the three subspecies of *M. abscessus.*

### The peaks of L29, L30, and hemophore-related protein in clinical isolates

The evaluation of L29, L30, and hemophore-related protein using 33 clinical strains of *M. abscessus* shows the peaks of 11 *M. abscessus* subsp. *abscessus* isolates at *m/z* 8780.9 as L29 and at *m/z* 9473.8 as hemophore-related protein, and the peaks of 20 *M. abscessus* subsp. *massiliense* isolates were detected at *m/z* 8766.9 as L29 and at *m/z* 9500.3 as hemophore-related protein. The remaining 2 isolates of *M. abscessus* subsp. *massiliense* were detected at *m/z* 8766.9 as L29 and at *m/z* 9473.8 as hemophore-related protein (Table 3). The condition of these evaluations was conducted in Middlebrook 7H11 Agar plates by MALDI-8020.

**Table 3:** The peaks of biokarker proteins in clinical isolates of *Mycobacterium abscessus.*

Subspecies	Strains	Average of L29	SE	Average of hemophore-related protein	SE
*M. abscessus* subsp. abscessus*	type strain	8781.0	0.21	9473.2	0.22
	M3	8780.9	0.16	9472.8	0.11
	M4	8780.2	0.19	9472.6	0.28
	M7	8781.2	0.23	9473.2	0.26
	M10	8780.8	0.33	9472.8	0.34
	M14	8780.3	0.30	9472.5	0.33
	M16	8780.9	0.28	9472.9	0.36
	M19	8780.7	0.37	9473.0	0.31
	M20	8780.3	0.25	9472.1	0.36
	M23	8780.6	0.28	9472.9	0.28
	M33	8779.5	0.22	9471.9	0.30
	M34	8780.5	0.20	9472.9	0.37
*M. abscessus* subsp. massiliense**	Type strain	8768.1	0.24	9502.4	0.35
	M5	8766.1	0.31	9500.2	0.30
	M6	8766.8	0.26	9501.3	0.39
	M8	8766.1	0.15	9500.3	0.09
	M11	8765.6	0.35	9500.3	0.35
	M13	8765.9	0.15	9500.2	0.12
	M15	8766.1	0.31	9500.3	0.33
	M26	8765.7	0.25	9499.7	0.04
	M27	8766.7	0.34	9501.5	0.27
	M28	8766.5	0.23	9500.8	0.37
	M29	8765.6	0.34	9500.3	0.37
	M30	8766.0	0.36	9500.3	0.34
	M32	8765.6	0.13	9499.8	0.23
	M36	8766.1	0.23	9500.4	0.20
	M37	8765.6	0.39	9499.8	0.30
	M38	8766.2	0.34	9500.4	0.35
	M39	8765.9	0.38	9500.2	0.26
	M40	8766.1	0.28	9500.4	0.40
	M43	8767.0	0.19	9501.4	0.31
	M44	8765.4	0.25	9499.7	0.29
	M45	8767.0	0.38	9501.2	0.37
	M41	8766.1	0.20	9472.6	0.21
	M42	8766.2	0.10	9472.8	0.08
*M. abscessus* subsp. bolletii*	Type strain	8784.5	0.43	9477.3	0.44

*The theoretical masses of L29 and hemophore-related protein are *m/z* 8780.9 and *m/z* 9473.8.

**The theoretical masses of L29 and hemophore-related protein are *m/z* 8766.9 and *m/z* 9500.3.

### Phylogenetic analysis and cluster analysis

**Figure 4:**
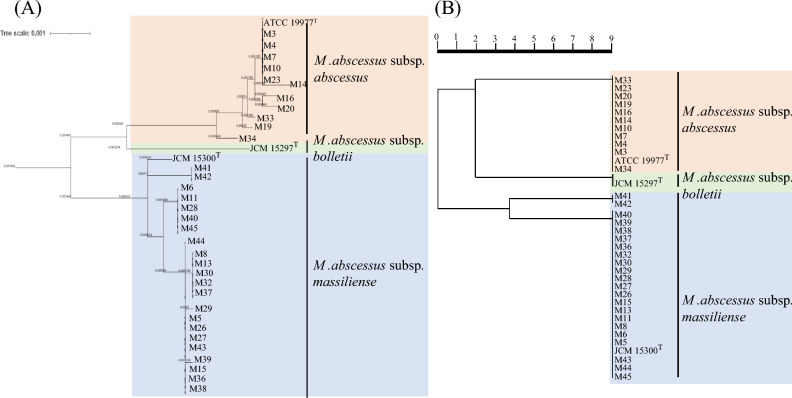
Phylogenetic tree of 33 clinical isolates of *M. abscessus* and three type strains of *M. abscessus*, including *M. abscessus* subsp. *abscessus*, *M. abscessus* subsp. *bolletii,* and *M. abscessus* subsp. *massiliense.* (**A**) The tree was constructed based on concatenated single-copy marker protein sequences predicted from genomes. (**B**) The tree was constructed by Strain Solution based on the biomarker proteins from the MALDI-MS data.

A genome-based phylogenetic tree was classified into the three groups of *M. abscessus* subsp. *abscessus, M. abscessus* subsp. *bolletii,* and *M. abscessus* subsp. *massiliense.* Furthermore, 2 isolates with peaks at *m/z* 9473.8 as hemophore-related protein shown in Table 3 were subclassified in the *M. abscessus* subsp. *massiliense* group (Fig. 4A).

A cluster analysis using the MALDI-MS data reveals that *M. abscessus* subsp. *abscessus, M. abscessus* subsp. *bolletii,* and *M. abscessus* subsp. *massiliense* are classified into the three groups (Fig. 4B). The phylogenic trees based on whole-genome and cluster analysis are identical.

## Discussion

MALDI-MS proteotyping is useful for accurate and rapid identification of *M. abscessus* subspecies, compared to sequencing using ANI, 16S rRNA, *rpoB*, *hsp65*, and *erm* genes or GenoType NTM-DR. While GenoType NTM-DR was developed for identifying *M. abscessus* subspecies and for determining resistance against clarithromycin and amikacin in 2016^18^, this method does not seem to be prevalent in clinical laboratories.

The prediction of the theoretical protein masses based on genomes of *M. abscessus* is useful for detecting the biomarker peaks using MALDI-MS. The theoretical protein mass database will accurately identify bacterial strains at the species to subspecies levels based in which correct identification for > 90% of measured spectra using MALDI-MS^29^. This approach can easily predict MALDI-MS spectra based on genome sequences from cultured and uncultured strains rather than experimentally acquired spectra. In this study, 19 detected peaks could be assigned with annotated proteins and 3 of 19 biomarker peaks, including ribosomal L29, L30 and hemophore-related protein, were screened as biomarkers for detecting subspecies of *M. abscessus*. Although we need to validate the identification for the other *Mycobacterium* in future, the closely related *Mycobacterium* species will not affect the identification of* M. abscessus* subspecies, because the amino acids of L29, L30 and hemophore-related protein are different.

Ribosomal protein L29, L30, and hemophore-related protein will be useful candidates as biomarkers for detecting subspecies of *M. abscessus*. Many of the peaks of MALDI- MS are derived from the ribosomal proteins, but it is difficult to extract and detect the protein fragments that require additional sample preparation for some microorganisms such as *Mycobacterium* spp.^30^. In the previous reports, the specific peaks of *M. abscessus* are distinct due to differences in sample preparation, mediums, and the instruments used^25^. In this study, all samples were crushed by a high-speed homogenizer and frozen according to the recommended methods in previous reports^22,25^. It has been reported that freezing the samples prior to MALDI-MS analysis effectively damages the bacterial cells for detecting MS peaks^31^. The peaks of L29, L30, and hemophore-related protein can be detected regardless of the mediums and the instruments.

In previous reports, four to seven peaks were used for distinguishing *M. abscessus* subspecies^20,22–26^, but we recommend a combination of three peaks: L29, L30, and hemophore-related protein. The three candidate proteins were screened by genome annotations and theoretical-protein-mass predictions. Suzuki et al.^25^ reported the peaks of *m/z* 8780.9 and *m/z* 9473.8 of *M. abscessus* subsp. *abscessus* and *M. abscessus* subsp. *bolletii* and the peaks of *m/z* 8766.9 and *m/z* 9500.3 of *M. abscessus* subsp. *massiliense.* The report also describes the peaks of *m/z* 4391.24 and *m/z* 4385.05, which are the divalent ion of the peaks of *m/z* 8780.9 and *m/z* 8766.9, respectively, for detecting *M. abscessus*. We newly developed the target peaks of L30, *m/z* 6765.9 and *m/z* 6795.9, for detecting *M. abscessus* subsp. *bolletii.*

Hemophore-related protein will be essential as a target peak. Previously, it has been reported that some isolates of *M. abscessus* subsp. *massiliense* has peaks of *m/z* 9473.82^25^ and *m/z* 9473.31^24^, which are similar to our finding for hemophore-related protein in *M. abscessus* subsp. *abscessus*. Using hemophore-related protein as a biomarker peak for distinguishing the subspecies will prevent misidentification.

For the early determination of effective therapy, it is necessary to distinguish the three *M.abscessus* subspecies’ susceptibilities to macrolides in routine testing. However, several isolates of clarithromycin-sensitive *M. abscessus* subsp. *abscessus* and clarithromycin-resistant *M. abscessus* subsp. *massiliense* with specific gene mutations were detected in a previous study^32^ as well as this study. Thus, the bacterial identification tests including MALDI-MS have a limitation in that they cannot accurately estimate drug susceptibility. On the other hand, drug susceptibility testing is not enough for the classification of strains harboring resistant genes. Therefore, the combination of MALDI-MS and drug susceptibility testing is important for the identification of *M. abscessus* subspecies in clinical laboratories.

This study has several limitations: first, this is a single-center study with a small number of samples. Second, the data from the clinical isolates of *M. abscessus* subsp. *bolletii* is missed in this study. It is necessary to confirm that the three biomarkers, including L29, L30 and hemophore-related protein, are useful for the identification of *M. abscessus* subspecies using more isolates obtained in the other hospital laboratories from different countries in future. Third, although the amino acid sequences of L29, L30 and hemophore-related protein were unique in *M. abscessus* subspecies, it is necessary to confirm the spectra of the other RGMs with different amino acid sequences and the same theoretical protein mass.

In conclusion, the detection of the peaks of L29, L30, and hemophore-related protein by MALDI-MS proteotyping will be useful for accurate and rapid identification of *M. abscessus*, compared to traditional methods of sequencing. The identification of *M. abscessus* by MALDI-MS combined with drug susceptibility testing will be the best way for an early decision on a course of treatment.

## Materials and methods

### Bacterial strains

The type strains of each subspecies were used to select biomarker candidates to distinguish the three subspecies of *M. abscessus* using MALDI-MS. The type strain of *M. abscessus* subsp. *abscessus* GTC 15115^T^ (= ATCC 19977^T^) was obtained from Gifu University Center for Conservation of Microbial Genetic Resource and the type strains of *M. abscessus* subsp. *bolletii* JCM 15297^T^ and *M. abscessus* subsp. *massiliense* JCM 15300^T^ were both obtained from the RIKEN BRC (Tsukuba, Ibaraki, Japan). Thirty-three clinical isolates of *M. abscessus* were obtained between June 2013 and October 2019 from 33 patients treated at Juntendo University Hospital in Japan. The isolates were cultured in Middlebrook 7H11 Agar plates (Becton, Dickinson-Diagnostic Systems) or 5% sheep blood agar (Becton, Dickinson-Diagnostic Systems) under aerobic conditions at 35 °C for 3 days.

### Drug susceptibility testing

Drug susceptibility was tested as described by the Clinical and Laboratory Standard Institute (CLSI) guidelines^28^. The antibiotic concentrations of clarithromycin, amikacin, and imipenem ranged from 0.063 to 128 μg/mL. The minimum inhibitory concentrations (MICs) of each antimicrobial agent were determined by broth microdilution methods using Muller Hinton broth and 96-well microtiter plates (Kohjin Bio, Co., Ltd. Saitama, Japan). The MICs of clarithromycin, amikacin, and imipenem were determined on the 5th day at an early reading time (ERT) and on the 14th day at a delayed reading time (LRT).

### Whole-genome sequencing

Genomic DNA of the 33 clinical *M. abscessus* isolates were extracted using DNeasy blood and tissue kits (Qiagen, Tokyo, Japan) and DNA libraries were prepared using Nextera XT DNA Library Prep Kit (Illumina, San Diego, CA). Their genomes were sequenced by Illumina MiSeq platform using v3 chemistry (600 cycles) and the summary of the assembly is shown in Supplementary Table S5. Raw reads of each isolate were trimmed and assembled using CLC Genomic Workbench version 10.0.1 using quality control and assembly tools with default settings (CLC bio, Aarhus, Denmark). Species identities of these isolates were determined using an ANI calculator^33^ and the sequences of 16S rRNA, *rpoB*, *hsp65*, and *erm* genes^12,15^. The ANI values and sequence identities of 16S rRNA, *rpoB*, *hsp65*, and *erm* genes were calculated and adopt the closest of the reference genomes of *M. abscessus* subsp. *abscessus* (ATCC 19977^T^, genome accession number GCF_000069185.1), *M. abscessus* subsp. *bolletii* (JCM 15297^T^, GCF_003609715.1), and *M. abscessus* subsp. *massiliense* (JCM 15300^T^, GCF_000497265.2)^27^. The mutations of *erm* and *rrl* genes were detected in silico using CLC Genomics Workbench^34^.

### Phylogenetic analysis

The genome completeness and contamination were assessed using CheckM2 v1.0.1 with lineage wf and default settings^35^. Phylogenetic trees were constructed based on concatenated single-copy marker protein sequences predicted from genomes using GTDB-Tk v2.2.6 software^36^ and visualized using iTol ver.6 (https://itol.embl.de/). The type strains of *M. abscessus* subsp. *abscessus* (ATCC 19977^T^, genome accession number GCF_000069185.1), *M. abscessus* subsp. *bolletii* (JCM 15297^T^, GCF_003609715.1), and *M. abscessus* subsp. *massiliense* (JCM 15300^T^, GCF_000497265.2) were used as the reference strains.

### Accession numbers

The whole-genome sequences of all 33 isolates have been deposited in the GenBank as accession number PRJDB15290.

### Calculation of the theoretical mass of *M. abscessus*

Theoretical masses of proteins encoded in genomes of *M. abscessus* were calculated for the following genomes as part of the development of a genomically predicted protein mass database of *Bacteria* and *Archaea*: *M. abscessus* subsp. *abscessus* (ATCC 19977^T^, GCF_000069185.1), *M. abscessus* subsp. *bolletii* (JCM 15297^T^, GCF_003609715.1), and *M. abscessus* subsp. *massiliense* (JCM 15300^T^, GCF_000497265.2)^29^*.* The genome sequences were obtained from the NCBI database (https://www.ncbi.nlm.nih.gov/). Gene prediction from the genomes was performed using Prodigal v2.6.3^37^. The calculation of the theoretical mass of individual gene products was performed by python scripts with consideration of the average [M + H]^+^ of all the gene products. For all amino-acid sequences, methionine loss was considered if the first amino acid at the N-terminal was “M” and the second amino acid was either “G”, “A”, “S”, “P”, “V”, “T”, or “C”^29^. Mature protein sequences were inferred using SignalP v5.0^38^ with command line flags “-org gram + ” for genomes.

### Bacterial sample preparation for MALDI-MS

Alpha-cyano-4-hydroxycinnamic acid (CHCA) was used as a matrix. To prepare this matrix solution, 10 mg of 4-CHCA was dissolved in 1 mL of the solvent consisting of 1% (v/v) trifluoroacetic acid, 35% (v/v) ethanol, 15% (v/v) acetonitrile, and milliQ water. A full loop of bacterial cells was dispersed in 200 μL of distilled water in a microtube and mixed with 800 μL of ethanol with zirconia beads. The suspensions were vortexed briefly and centrifuged at 15,000 *g* for 2 min. The pellets were then dried for 5 min. After freezing the tubes at − 80 °C at least 1 h, the pellets were resuspended in MilliQ water, crushed using a Fast Prep 24 apparatus (Funakoshi Co., Ltd.) for a total of 3 min (9 times for 20 s), and centrifuged at 15,000 *g* for 2 min. Supernatants were analyzed by MALDI-MS according to the manufacturer’s instruction.

### MALDI-MS measurement

MALDI-MS measurements were performed in positive linear mode using MALDI-8020 RUO (Shimadzu Corporation, Japan) and Microflex LT/SH (Bruker Daltonik) equipped with a 200 Hz Nd: YAG laser (355 nm) and 60 Hz nitrogen laser (337 nm), respectively.

Before the sample analysis, the MALDI-MS instrument was mass-calibrated externally using 6 peaks with *m/z* 4365.4, 5381.4, 6411.6, 7274.0, 8369.8, and 10,300.1 from *Escherichia coli* DH5α. More than five individual mass spectra were acquired for each bacterial extract in the range of *m/z* 2000–20,000 and self-calibrated using three ribosomal protein peaks with *m/z* 4371.3, 6310.2, and 9348.8, which were commonly detected from corresponding type strains of the three subspecies of *M. abscessus*. Peak assignment was carried out using eMSTAT Solution™ software (Shimadzu Corp.). The peaks were assigned using a comparison with the calculated masses of genome sequenced type strains of *M. abscessus* subsp. *abscessus, M. abscessus* subsp. *bolletii,* and *M. abscessus* subsp. *massiliense*.

### Cluster analysis

For biomarker validation, 36 *M. abscessus* isolates including three subspecies were analyzed by MALDI-MS. Four mass spectra were acquired for each strain, and peak lists were extracted from those mass spectra, considering the peak intensity and reproducibility. Biomarker analysis software Strain Solution™ was used to prepare a binary matrix.

### Ethical statement

This study was approved by the Ethical Committee of Juntendo University (approval number: E21-0232).

### Supplementary Information

Supplementary Tables.

## Data Availability

All data generated or analyzed during this study are included in this published article. The sequence data generated in this study have been submitted to the DDBJ database (http://getentry.ddbj.nig.ac.jp/) under the accession numbers PRJDB15290.
