# Supplementary material for: Identification of Mycobacterium abscessus using the peaks of ribosomal protein L29, L30 and hemophore-related protein by MALDI-MS proteotyping
Source: Sci Rep. 2024 May 16;14:11187. doi: 10.1038/s41598-024-61549-7 (PMC11099050; doi:10.1038/s41598-024-61549-7)
Supplement: Supplementary file 1 — Supplementary Tables. [file 41598_2024_61549_MOESM1_ESM.pdf]

**Table S1** Subspecies identification based on ANI, 16S rRNA, *rpoB*, *hsp65*, and *erm*(41) genes using whole genome sequencing

| Subspecies[a] | ANI | | | 16S rRNA | | | *rpoB* | | | *hsp65* | | | *erm*(41) |
|---|---|---|---|---|---|---|---|---|---|---|---|---|---|
| | *M. abscessus* subsp. *abscessus*[T] | *M. abscessus* subsp. *bolletii*[T] | *M. abscessus* subsp. *massiliense*[T] | *M. abscessus* subsp. *abscessus*[T] | *M. abscessus* subsp. *bolletii*[T] | *M. abscessus* subsp. *massiliense*[T] | *M. abscessus* subsp. *abscessus*[T] | *M. abscessus* subsp. *bolletii*[T] | *M. abscessus* subsp. *massiliense*[T] | *M. abscessus* subsp. *abscessus*[T] | *M. abscessus* subsp. *bolletii*[T] | *M. abscessus* subsp. *massiliense*[T] | |
| *M. abscessus* subsp. *abscessus* | 91.5-99.9 | 76.4-78.2 | 75.3-77.4 | 100 | 100 | 100 | 100 | 96.58 | 97.31 | 100 | 98.5 | 98.75 | full |
| *M. abscessus* subsp. *massiliense* | 75.9-78.7 | 73-74.8 | 89.1-95.57 | 100 | 100 | 100 | 97.31 | 98.29 | 100 | 98.75 | 99.25 | 100 | deletion |

[a] clinical isolates of *M. abscessus* subspecies

**TABLE S2** Summary of the characteristics of 33 isolates of *Mycobacterium abscessus*

| Isolates | Isolation date | Subspecies | MIC (μg/ml) | | | | Drug resistant mutations | |
|---|---|---|---|---|---|---|---|---|
| | | | Clarithromycin | | Amikacin | Imipenem | | |
| | | | day5 | day14 | day5 | day5 | *erm*(41) | *rrl* |
| M3 | March 2014 | *M. abscessus* subsp. *abscessus* | 1 | 128 | 8 | 16 | T28 | - |
| M4 | May 2017 | *M. abscessus* subsp. *abscessus* | 0.25 | >128 | 16 | 8 | T28 | - |
| M5 | July 2015 | *M. abscessus* subsp. *massiliense* | >128 | >128 | 16 | 16 | T28 | a2058c |
| M6 | May 2017 | *M. abscessus* subsp. *massiliense* | ≤0.06 | ≤0.06 | 8 | 8 | T28 | - |
| M7 | August 2016 | *M. abscessus* subsp. *abscessus* | 0.5 | 128 | 8 | 16 | T28 | - |
| M8 | April 2016 | *M. abscessus* subsp. *massiliense* | ≤0.06 | ≤0.06 | 8 | 8 | T28 | - |
| M10 | June 2014 | *M. abscessus* subsp. *abscessus* | 0.25 | 128 | 8 | 8 | T28 | - |
| M11 | May 2017 | *M. abscessus* subsp. *massiliense* | ≤0.06 | 0.125 | 8 | 16 | T28 | - |
| M13 | May 2017 | *M. abscessus* subsp. *massiliense* | ≤0.06 | ≤0.06 | 16 | 16 | T28 | - |
| M14 | December 2013 | *M. abscessus* subsp. *abscessus* | 0.125 | 16 | 8 | 8 | T28 | - |
| M15 | October 2015 | *M. abscessus* subsp. *massiliense* | >128 | >128 | 8 | 2 | T28 | a2058c |
| M16 | April 2016 | *M. abscessus* subsp. *abscessus* | 0.25 | 4 | 16 | 4 | T28 | - |
| M19 | April 2017 | *M. abscessus* subsp. *abscessus* | ≤0.06 | ≤0.06 | 8 | 16 | C28 | - |
| M20 | June 2013 | *M. abscessus* subsp. *abscessus* | 0.125 | 32 | 4 | 4 | T28 | - |
| M23 | February 2012 | *M. abscessus* subsp. *abscessus* | >128 | >128 | 8 | 8 | T28 | - |
| M26 | May 2016 | *M. abscessus* subsp. *massiliense* | ≤0.06 | ≤0.06 | 16 | 16 | T28 | - |
| M27 | May 2017 | *M. abscessus* subsp. *massiliense* | ≤0.06 | ≤0.06 | 8 | 16 | T28 | - |
| M28 | October 2016 | *M. abscessus* subsp. *massiliense* | ≤0.06 | ≤0.06 | 4 | 4 | T28 | - |
| M29 | November 2016 | *M. abscessus* subsp. *massiliense* | ≤0.06 | 0.25 | 8 | 16 | T28 | - |
| M30 | April 2016 | *M. abscessus* subsp. *massiliense* | ≤0.06 | ≤0.06 | 16 | 16 | T28 | - |
| M32 | October 2016 | *M. abscessus* subsp. *massiliense* | ≤0.06 | ≤0.06 | 16 | 8 | T28 | - |
| M33 | February 2018 | *M. abscessus* subsp. *abscessus* | 0.125 | 128 | 8 | 8 | T28 | - |
| M34 | June 2018 | *M. abscessus* subsp. *abscessus* | ≤0.06 | ≤0.06 | 8 | 8 | C28 | - |

| | | | | | | | | |
|---|---|---|---|---|---|---|---|---|
| M36 | December 2018 | *M. abscessus* subsp. *massiliense* | ≤0.06 | ≤0.06 | 8 | 16 | T28 | - |
| M37 | November 2018 | *M. abscessus* subsp. *massiliense* | ≤0.06 | ≤0.06 | 8 | 8 | T28 | - |
| M38 | December 2018 | *M. abscessus* subsp. *massiliense* | ≤0.06 | ≤0.06 | 0.125 | ≤0.06 | T28 | - |
| M39 | March 2018 | *M. abscessus* subsp. *massiliense* | ≤0.06 | ≤0.06 | 8 | 4 | T28 | - |
| M40 | January 2019 | *M. abscessus* subsp. *massiliense* | ≤0.06 | ≤0.06 | 8 | 8 | T28 | - |
| M41 | June 2019 | *M. abscessus* subsp. *massiliense* | ≤0.06 | ≤0.06 | 8 | 8 | T28 | - |
| M42 | February 2018 | *M. abscessus* subsp. *massiliense* | ≤0.06 | ≤0.06 | 2 | 2 | T28 | - |
| M43 | June 2019 | *M. abscessus* subsp. *massiliense* | ≤0.06 | ≤0.06 | 8 | 8 | T28 | - |
| M44 | July 2019 | *M. abscessus* subsp. *massiliense* | ≤0.06 | ≤0.06 | 8 | 4 | T28 | - |
| M45 | October 2019 | *M. abscessus* subsp. *massiliense* | ≤0.06 | ≤0.06 | 8 | 8 | T28 | - |

**TABLE S3** Annotated peaks of type strains of *Mycobacterium abscessus* cultured in 5% sheep blood agar by MALDI-8020

| Biomarker proteins | Subspecies | | | | | | | | | | | |
|---|---|---|---|---|---|---|---|---|---|---|---|---|
| | *Mycobacterium abscessus* subsp. *abscessus* ATCC 19977$^T$ | | | | *Mycobacterium abscessus* subsp. *bolletii* JCM 15297$^T$ | | | | *Mycobacterium abscessus* subsp. *massiliense* JCM 15300$^T$ | | | |
| | Calculated masses *(m/z)* | Average | SE | Peak numbers (n=5) | Calculated masses *(m/z)* | Average | SE | Peak numbers (n=5) | Calculated masses *(m/z)* | Average | SE | Peak numbers (n=5) |
| L29 | 8780.9 | 8780.4 | 0.19 | 5 | 8780.9 | 8780.6 | 0.17 | 5 | 8766.9 | 8767.1 | 0.23 | 5 |
| L30 | 6795.9 | 6795.8 | 0.09 | 5 | 6765.9 | 6766.4 | 0.04 | 5 | 6795.9 | 6795.9 | 0.22 | 5 |
| hemophore-related protein | 9473.8 | 9472.4 | 0.07 | 5 | 9473.8 | 9472.6 | 0.18 | 5 | 9500.3 | 9501.0 | 0.29 | 5 |

**TABLE S4** Annotated peaks type strains of *Mycobacterium abscessus* cultured in 7H11 agar analyzed by Microflex LT/SH

| Biomarker proteins | Subspecies | | | | | | | | | | | |
|---|---|---|---|---|---|---|---|---|---|---|---|---|
| | *Mycobacterium abscessus* subsp. *abscessus* ATCC 19977$^T$ | | | | *Mycobacterium abscessus* subsp. *bolletii* JCM 15297$^T$ | | | | *Mycobacterium abscessus* subsp. *massiliense* JCM 15300$^T$ | | | |
| | Calculated masses *(m/z)* | Average | SE | Peak numbers (n=5) | Calculated masses *(m/z)* | Average | SE | Peak numbers (n=5) | Calculated masses *(m/z)* | Average | SE | Peak numbers (n=5) |
| L29 | 8780.9 | 8781.0 | 0.28 | 5 | 8780.9 | 8780.6 | 0.47 | 5 | 8766.9 | 8768.3 | 0.30 | 5 |
| L30 | 6795.9 | 6796.6 | 0.10 | 5 | 6765.9 | 6767.2 | 0.29 | 5 | 6795.9 | 6797.4 | 0.23 | 5 |
| hemophore-related protein | 9473.8 | 9473.1 | 0.23 | 5 | 9473.8 | 9472.9 | 0.37 | 5 | 9500.3 | 9502.1 | 0.38 | 5 |

**TABLE S5** Assembly sammary report of 33 *M. abscessus* isolates

| Strains | Accession No. | Reads (bp) | Coverage | Contigs | Completeness | Contamination | Contig_N50 |
|---|---|---|---|---|---|---|---|
| M3 | SAMD00579515 | 657495000 | 131.0 | 99 | 100 | 0.35 | 124111 |
| M4 | SAMD00579516 | 198122000 | 39.5 | 106 | 100 | 0.42 | 98096 |
| M5 | SAMD00579517 | 190007000 | 38.5 | 67 | 100 | 0.27 | 131541 |
| M6 | SAMD00579518 | 157454000 | 31.9 | 91 | 100 | 0.18 | 118404 |
| M7 | SAMD00579519 | 243205000 | 48.4 | 84 | 100 | 0.69 | 184713 |
| M8 | SAMD00579520 | 186904000 | 37.9 | 100 | 100 | 0.18 | 97993 |
| M10 | SAMD00579521 | 142809000 | 28.4 | 130 | 100 | 0.35 | 94441 |
| M11 | SAMD00579522 | 207484000 | 42.1 | 174 | 99.97 | 0.27 | 134295 |
| M13 | SAMD00579523 | 180423000 | 36.6 | 110 | 100 | 0.24 | 103151 |
| M14 | SAMD00579524 | 225807000 | 45.0 | 92 | 99.99 | 0.38 | 105767 |
| M15 | SAMD00579525 | 248498000 | 50.4 | 72 | 100 | 0.74 | 132833 |
| M16 | SAMD00579526 | 168764000 | 33.6 | 114 | 99.99 | 0.63 | 98330 |
| M19 | SAMD00579527 | 214400000 | 42.7 | 90 | 100 | 0.74 | 100560 |
| M20 | SAMD00579528 | 134439000 | 26.8 | 171 | 100 | 0.43 | 64694 |
| M23 | SAMD00579529 | 258753000 | 51.5 | 74 | 100 | 0.3 | 117901 |
| M26 | SAMD00579530 | 219897000 | 44.6 | 91 | 100 | 1.52 | 116993 |
| M27 | SAMD00579531 | 147412000 | 29.9 | 229 | 99.73 | 0.37 | 67341 |
| M28 | SAMD00579532 | 183780000 | 37.3 | 166 | 100 | 0.18 | 99111 |
| M29 | SAMD00579533 | 239480000 | 48.6 | 114 | 100 | 0.31 | 109330 |

| | | | | | | | |
|---|---|---|---|---|---|---|---|
| M30 | SAMD00579534 | 227198000 | 46.1 | 75 | 100 | 0.19 | 136418 |
| M32 | SAMD00579535 | 225434000 | 45.7 | 99 | 99.99 | 0.19 | 112641 |
| M33 | SAMD00579536 | 196673000 | 39.2 | 119 | 100 | 0.55 | 107106 |
| M34 | SAMD00579537 | 173890000 | 34.6 | 137 | 100 | 0.25 | 120369 |
| M36 | SAMD00579538 | 247069000 | 50.1 | 71 | 100 | 0.29 | 132334 |
| M37 | SAMD00579539 | 218580000 | 44.3 | 53 | 100 | 0.23 | 145475 |
| M38 | SAMD00579540 | 232395000 | 47.1 | 94 | 100 | 0.42 | 127101 |
| M39 | SAMD00579541 | 252511000 | 51.2 | 78 | 100 | 0.23 | 142937 |
| M40 | SAMD00579542 | 247147000 | 50.1 | 127 | 100 | 0.26 | 118863 |
| M41 | SAMD00579543 | 182194000 | 36.9 | 123 | 100 | 0.36 | 121499 |
| M42 | SAMD00579544 | 264464000 | 53.6 | 79 | 100 | 0.82 | 128982 |
| M43 | SAMD00579545 | 189489000 | 38.4 | 95 | 100 | 0.22 | 130554 |
| M44 | SAMD00579546 | 230135000 | 46.7 | 84 | 100 | 0.21 | 118308 |
| M45 | SAMD00579547 | 398175000 | 80.7 | 101 | 100 | 0.39 | 152882 |
